# Validation of resting full-cycle ratio and diastolic pressure ratio with [^15^O]H_2_O positron emission tomography myocardial perfusion

**DOI:** 10.1007/s00380-023-02356-4

**Published:** 2024-02-17

**Authors:** Jorge Dahdal, Frank Bakker, Johan Svanerud, Ibrahim Danad, Roel S. Driessen, Pieter G. Raijmakers, Hendrik J. Harms, Adriaan A. Lammertsma, Tim P. van de Hoef, Yolande Appelman, Niels van Royen, Paul Knaapen, Guus A. de Waard

**Affiliations:** 1 Department of Cardiology, Amsterdam University Medical Center, De Boelelaan 1117, 1081 HV Amsterdam, The Netherlands; 2 Department of Cardiology, Hospital Del Salvador, Salvador 364, 7500922 Santiago, Chile; 3 Coroventis Research AB, Ulls Väg 29A, 75651 Uppsala, Sweden; 4 Utrecht University Medical Center, Heidelberglaan 100, 3584 CX Utrecht, The Netherlands; 5 Department of Radiology and Nuclear Medicine, VU University Medical Center, De Boelelaan 1117, 1081 HV Amsterdam, The Netherlands; 6 Clinical Institute, Aarhus University, Palle Juul-Jensens Blvd. 82, 8200 Aarhus, Denmark; 7 Department of Cardiology, Radboud University Medical Center, Geert Grooteplein Zuid 10, 6525 GA Nijmegen, The Netherlands

**Keywords:** NHPR, RFR, dPR, FFR, Positron emission tomography

## Abstract

Fractional flow reserve (FFR) and instantaneous wave-free ratio (iFR) are invasive techniques used to evaluate the hemodynamic significance of coronary artery stenosis. These methods have been validated through perfusion imaging and clinical trials. New invasive pressure ratios that do not require hyperemia have recently emerged, and it is essential to confirm their diagnostic efficacy. The aim of this study was to validate the resting full-cycle ratio (RFR) and the diastolic pressure ratio (dPR), against [^15^O]H_2_O positron emission tomography (PET) imaging. A total of 129 symptomatic patients with an intermediate risk of coronary artery disease (CAD) were included. All patients underwent cardiac [^15^O]H_2_O PET with quantitative assessment of resting and hyperemic myocardial perfusion. Within a 2 week period, coronary angiography was performed. Intracoronary pressure measurements were obtained in 320 vessels and RFR, dPR, and FFR were computed. PET derived regional hyperemic myocardial blood flow (hMBF) and myocardial perfusion reserve (MPR) served as reference standards. In coronary arteries with stenoses (43%, 136 of 320), the overall diagnostic accuracies of RFR, dPR, and FFR did not differ when PET hyperemic MBF < 2.3 ml min^−1^ (69.9%, 70.6%, and 77.1%, respectively) and PET MPR < 2.5 (70.6%, 71.3%, and 66.9%, respectively) were considered as the reference for myocardial ischemia. Non-significant differences between the areas under the receiver operating characteristic (ROC) curve were found between the different indices. Furthermore, the integration of FFR with RFR (or dPR) does not enhance the diagnostic information already achieved by FFR in the characterization of ischemia via PET perfusion. In conclusion, the novel non-hyperemic pressure ratios, RFR and dPR, have a diagnostic performance comparable to FFR in assessing regional myocardial ischemia. These findings suggest that RFR and dPR may be considered as an FFR alternative for invasively guiding revascularization treatment in symptomatic patients with CAD.

**Supplementary Information:**

The online version contains supplementary material available at 10.1007/s00380-023-02356-4.

## Introduction

In patients with chronic coronary disease, percutaneous coronary interventions (PCI) guided by fractional flow reserve (FFR) improves symptoms, quality of life and is a cost-effective strategy [1]. Currently, the physiological approach with FFR is recommended by clinical guidelines to invasively identify the hemodynamic significance of coronary stenosis and guide revascularization [2]. Conceptually, FFR measures the ratio of maximal myocardial blood flow in the presence of stenosis to the maximal theoretical blood flow in the absence of that stenosis, utilizing hyperemic agents. However, the use of hyperemic agents like intravenous adenosine might provoke adverse effects like transient dyspnea, chest pain, flushing, rhythm disturbances, and hypotension [3, 4]. Novel indices evaluating the pressure loss across the stenosis in specific portions of the cardiac cycle during resting conditions have emerged as an alternative approach to assess the functional severity of coronary stenoses, avoiding the side effects of hyperemic agents. The instantaneous wave-free ratio (iFR), a distal to aortic coronary pressure ratio during the wave-free period, is the only non-hyperemic pressure ratio (NHPR) validated with clinical outcomes in randomized controlled trials and also with [^15^O]H_2_O positron emission tomography (PET) quantitative myocardial perfusion. [3–5] With the advent of this new evidence, the latest guidelines recommend iFR-guided revascularization in chronic coronary syndromes, with the same level and strength of evidence as FFR [2]. Currently, the utilization of the iFR algorithm is limited to proprietary software available from a single vendor (Philips Volcano, San Diego, CA, USA). Two other NHPRs that have emerged use different segments of the cardiac cycle: the full-cycle ratio (RFR) and the diastolic pressure ratio (dPR). These two indexes have shown good diagnostic accuracy when FFR is used as a reference (87% for RFR and 88% for dPR) [6]. Also, the RFR and dPR correlate excellently with iFR. [6–8] With respect to the ability of dPR and RFR to evaluate myocardial ischemia, only one PET perfusion study has been conducted using ^13^N-ammonia as a PET tracer [6]. Unfortunately, given the incomplete and non-linear extraction rate of ^13^N-ammonia by myocardial cells during maximal hyperemia, tracer-kinetics models with strong assumptions are required to quantitatively estimate perfusion [9, 10]. Because of its favorable pharmacokinetic characteristics [^15^O]H_2_O PET is considered the gold standard non-invasive measurement to determine myocardial blood flow (MBF) [11].

Given the need to validate other non-hyperemic pressure indexes, the aim of this study was to test the diagnostic performance of RFR and dPR in detecting hemodynamically significant coronary stenoses, using quantitative myocardial perfusion derived from [^15^O]H_2_O PET as a reference and to assess the correlation of these indices with FFR.

## Methods

### Study population

This is a PACIFIC-1 sub-study (NCT01521468) [12]. In brief, the PACIFIC-1 study included 208 stable symptomatic patients with suspected coronary artery disease and an intermediate pre-test probability (defined by Diamond and Forrester criteria) who were referred for invasive coronary angiography. All patients underwent [^15^O]H_2_O PET myocardial perfusion imaging with quantitative myocardial blood flow assessed. Subsequently, invasive coronary angiography was carried out within two weeks following the PET scan. During the coronary angiography, intracoronary physiological measurements were performed in the three major coronary arteries, if anatomically possible. Exclusion criteria for the PACIFIC study included a previous history of myocardial infarction, prior coronary revascularization, ventricular arrhythmias, heart failure, estimated left ventricular ejection fraction < 50%, atrial fibrillation, second or third degree atrioventricular block, renal insufficiency, and contraindication for the use of adenosine.

For the present sub-study, the same study population of 129 patients from an earlier sub-study was used, in whom invasive resting pressure measurements were available [5].

This study was conducted in accordance with the declaration of Helsinki. The study protocol was approved by the Medical Ethics Committee of the VU University Medical Center, and all participants provided written informed consent.

### Invasive coronary angiography

Invasive coronary angiography was performed according to our institutional protocol using either the radial or femoral artery approach. Before angiography, 200–300 µg nitroglycerin was administered intracoronary. Using quantitative coronary angiography (CAAS II, Pie Medical, Maastricht, The Netherlands) in two orthogonal directions per coronary artery, diameter stenosis % was calculated by a single analyst (GdW). Pressure ratios were measured using a 0.014-inch pressure sensor tipped guide wire (Philips Volcano, San Diego, CA, USA) introduced through a 5-F or 6-F guiding catheter, with pressure recording normalized at the tip of the guiding catheter artery and then advanced and placed in the distal part of the artery. Coronary arteries with a chronic total or subtotal occlusion were not evaluated. Distal and aortic pressure traces were recorded at a sample rate of 200 Hz, containing at least ten beats, both at rest and in hyperemia. To induce maximal coronary hyperemia, intracoronary (150 µg) or intravenous (140 µg · kg^−1^ · min^−1^) adenosine infusion was used. After the physiological measurements, pressure drift was recorded. In case of significant drift (> 2 mmHg), the measurements were either repeated or corrected during offline analysis of FFR, dPR, and RFR.

### Pressure index calculation

**Fig. 1:**
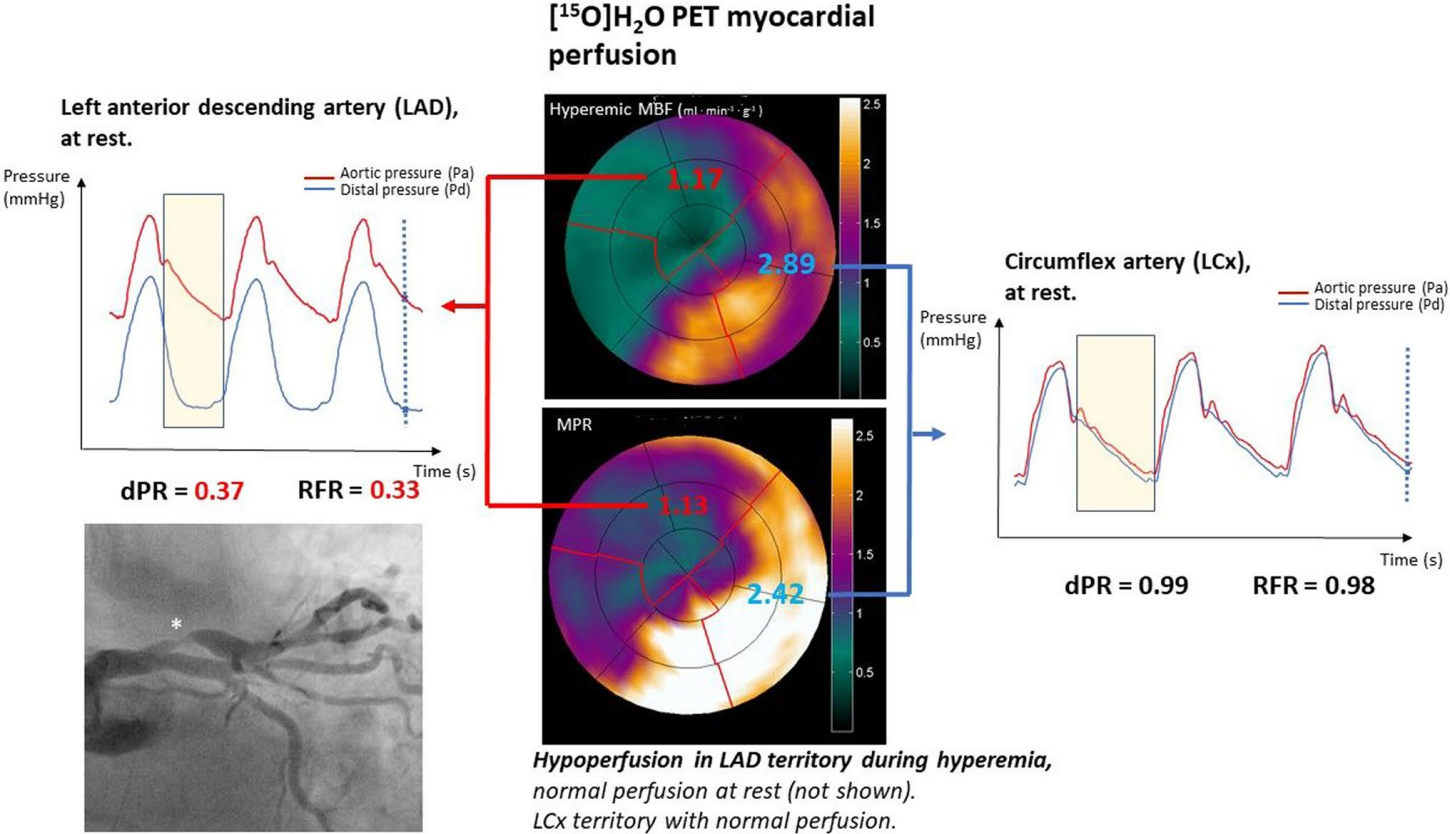
Results of [^15^O]H_2_O PET myocardial perfusion in hyperemia in a 75-year-old woman with suspected CAD. In the center, PET vessel-specific hyperemic myocardial blood flow, and myocardial perfusion reserve. Ischemic regional myocardial blood flow and perfusion reserve values were observed in the LAD territory, whilenormal perfusion was demostrated in the LCx territory. The patient had a normal perfusion during rest (not shown). In the left panel, the aortic and distal rest intracoronary pressure of the LAD are plotted. The yellow rectangle represents the period in which dPR is computed (ratio between the mean distal and the mean aortic pressure during the diastolic period). RFR is an immediate pressure ratio, the lowest during the whole cardiac cycle; represented by the dashed blue line (RFR is the average of this instantaneous ratio in 5 cardiac cycles). Abnormal results of dPR and RFR in LAD were concordant with results of the PET scan. The right panel shows a pressure recording for the LCx, with normal pressure ratios in this vessel. In the coronary angiogram the white asterisk highlights a severe proximal lesion of the LAD. *PET *Positron emission tomography, *RFR *resting full-cycle ratio, *dPR *diastolic pressure ratio, *FFR *fractional flow reserve, *MBF *myocardial blood flow, *MPR *myocardial perfusion reserve

All pressure indices were calculated by the analysis of the aortic (Pa) and distal (Pd) pressure trace. These indices (RFR, dPR, and FFR) are ratios between the distal and the aortic pressure in a specific segment or during the whole cardiac cycle. FFR was computed as the ratio between hyperemic Pd and Pa [5]. Calculations of RFR and dPR were done in a core laboratory, using an automated algorithm developed in dedicated proprietary software (Coroventis Research AB, Uppsala, Sweden). Indices were computed by a single analyst (JS) blinded to any other clinical information from the participants including coronary angiograms, PET result, the hyperemic pressure traces, and FFR results. Before calculations, the pressure drift and the temporal offset of the Pa and Pd pressure waveforms were equalized. The instantaneous Pd/Pa ratio was smoothed using an adaptive filter. dPR was defined as the mean resting Pd/Pa over the entire diastole, averaged from 5 consecutive cardiac cycles. RFR was defined as the lowest instantaneous resting Pd/Pa value in the cardiac cycle, averaged from 5 consecutive cardiac cycles (Fig. 1). Based on previous studies, we used $\le$ 0.89 as the threshold for RFR [13, 14] and dPR [6, 8]. FFR $\le$ 0.80 was considered to be indicative of a hemodynamically significant stenosis [2].

### Positron emission tomography acquisition

A hybrid PET-CT scanner (Philips Gemini TF 64, Philips Healthcare, Best, The Netherlands) using 370 MBq of [^15^O]H_2_O as tracer during resting and hyperemic conditions, was used to scan all the patients included. The [^15^O]H_2_O PET protocol, image acquisition, and regional vessel-specific quantification of myocardial MBF have been described previously [15]. To verify that the location of the stenoses interrogated with physiological ratios corresponded with the PET perfusion, PET scans were reviewed together with the coronary angiogram by two image analysts (RD and GdW) who were blinded to the physiological data obtained. Regional PET-derived hyperemic myocardial blood flow (MBF) and myocardial perfusion reserve (MPR), defined as the ratio between hyperemic and resting MBF with a ischemic threshold of < 2.3 ml · min^−1^ · g^−1^ and < 2.5, respectively, were used for statistical analysis (Fig. 1). These predefined values were identified as the most accurate cutoff based on previous literature [12, 16].

### Statistical analysis

Categorical variables were presented as numbers and proportions. Continuous variables were expressed as mean with standard deviation (SD) or median with interquartile range (IQR) according to their distributions. Data distribution was visually assessed with histograms of the variables. Data were analyzed on a per-patient basis only for clinical characteristics and on a per-vessel basis for physiological index analysis. The primary analysis was done in patients with coronary artery disease and a secondary analysis was conducted in all the vessels studied including coronary arteries without ≥ 30% diameter stenosis. Spearman correlation coefficients were used to estimate the correlations between non-normally distributed quantitative variables. The diagnostic performance of the invasive physiological indices was presented with sensitivity, specificity, positive predictive value, negative predictive value, and diagnostic accuracy, using regional PET derived hyperemic myocardial blood flow (hMBF) and MPR, as a reference. For this analysis, generalized estimating equations (GEE) were used to correct for potential correlation secondary to repeated measurements in the same patients (multiple vessels were studied in each patient). An independent working correlation matrix was used for these models [17]. Diagnostic accuracy of the physiological indices was compared using GEE models with an indicator for the index as the independent variable. Bonferroni correction for multiple comparison was applied. Cohen’s kappa coefficient (k) was used to report the level of agreement between the indices. Receiver operating characteristic (ROC) curves were generated for each physiological index to identify the presence of an impaired PET hMBF and also for MPR. The areas under the curve of the ROC curve of the different intracoronary ratios were compared using DeLong's test. Differences in the clinical characteristic across the subgroups defined by FFR and NHPR were done using Chi-Square and ANOVA. For assessing the added diagnostic information provided by the combined use of binary FFR and NHPR to predict ischemia defined by PET hMBF, a mixed-effect multiple logistic regression was used. The nested models performance was tested using likelihood ratio test (LRT). Two-sided *p* value < 0.05 were considered statistically significant. Statistical analysis was performed by JD. SPSS version 22.0 (SPSS, Chicago, Illinois) and MedCalc Statistical Software V. 20.115 (MedCalc Software Bvba, Ostend, Belgium) were used for statistical analysis.

## Results

### Baseline characteristics, coronary physiology, and PET results

**Fig. 2:**
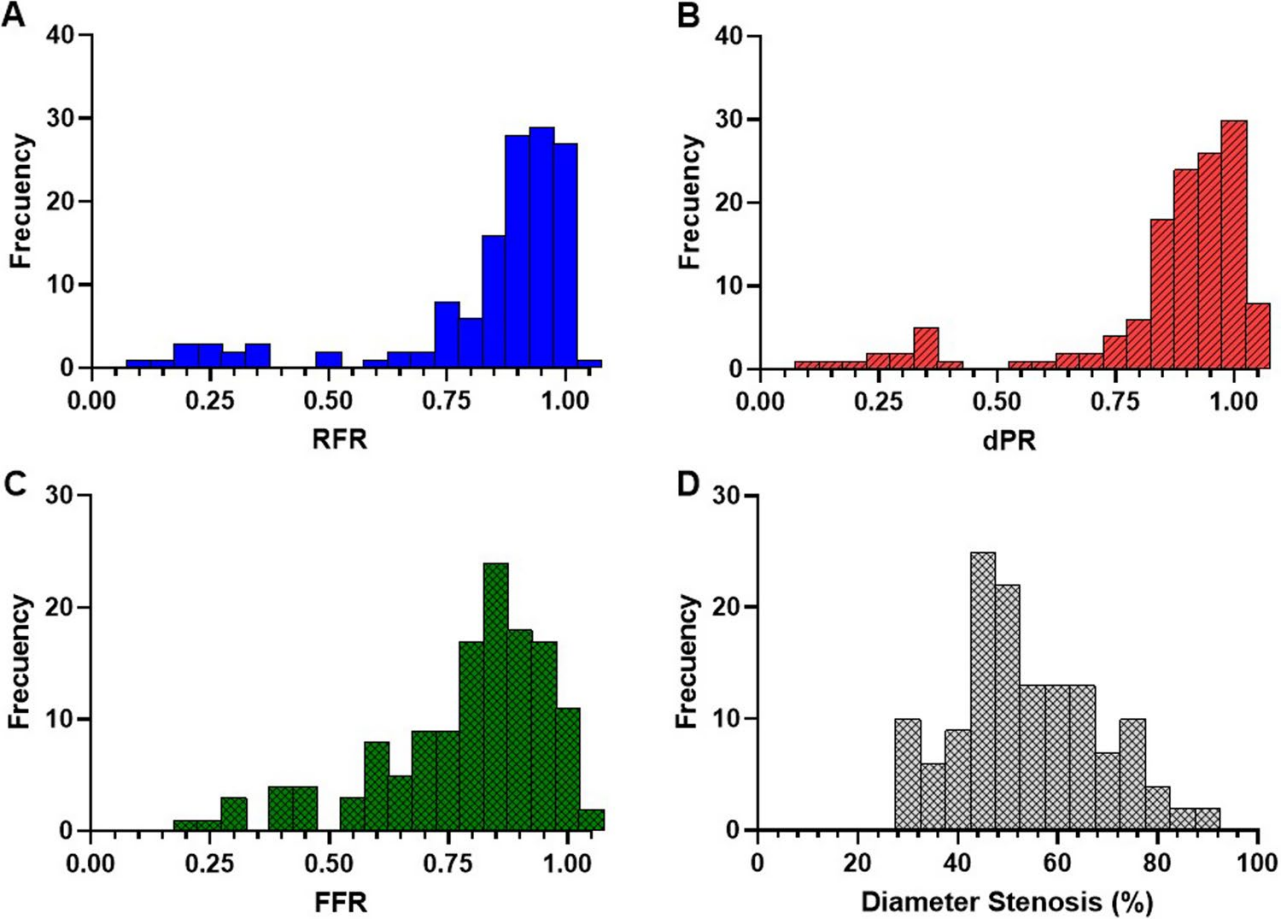
Frequency distribution of the physiological indices and diameter of coronary stenoses (*N* = 136). Histograms of the distributions of **A** RFR, **B** dPR, **C** FFR, and **D** diameter stenoses. Only in vessels with ≥ 30% diameter stenosis. *RFR *resting full-cycle ratio, *dPR *diastolic pressure ratio, *FFR *fractional flow reserve

Table 1 shows baseline patient characteristics. The mean age was 58 ± 8.6 years, and 83 patients (64%) were male. From the 320 vessels studied, 136 (43%) showed coronary stenosis. 64% of the stenoses were located in the left anterior descending (LAD) artery. The mean diameter stenosis was 54 ± 14%. In the coronary vessels with a stenosis, the median (IQR) values of FFR, RFR, and dPR were 0.84 (0.70–0.91), 0.91 (0.82–0.97) and 0.92 (0.84–0.98), respectively. [^15^O]H_2_O PET summary values are described in Table 2. The distribution of invasive physiological indices and angiographic lesion severity of patients with stenoses is shown in Fig. 2. The majority of the stenoses were angiographically moderate, but severe stenoses were also present (18% of stenosis). With regard to pressure indices distribution, FFR was more widely spread out than NHPRs that were more left skewed.

**Table 1:** Baseline characteristics on per-patients basis (*n* = 129)

Variables	
Male sex	83 (64%)
Age (years)	58 ± 8.6
Body mass index (BMI, kg/m2)	27 ± 3.6
Risk factors	
Current or ex-smoker	60 (47%)
Diabetes mellitus	20 (16%)
Hypertension	60 (47%)
Hypercholesterolemia	48 (37%)
Family history of coronary artery disease	66 (51%)

**Table 2:** Angiographic, functional, and PET characteristics of the studied vessels, only stenosis included (*N* = 136)

Variables	
Diameter stenosis (%)	53.9 ± 14.2
Coronary artery assessed	
Right coronary artery	36 (26%)
Left anterior descending artery	64 (48%)
Circumflex artery	36 (26%)
Physiological measurements	
RFR	0.91 (0.82–0.97)
dPR	0.92 (0.84–0.98)
FFR	0.84 (0.70–0.91)
PET results	
Hyperemic MBF (ml · min^−1^ · g^−1^)	2.32 ± 0.9
MPR	2.72 ± 0.9

*RFR* resting full-cycle ratio, *dPR* diastolic pressure ratio, *RFR *fractional flow reserve, *PET *positron emission tomography

### Physiological indices and positron emission tomography quantitative perfusion in the coronary stenoses group

**Fig. 3:**
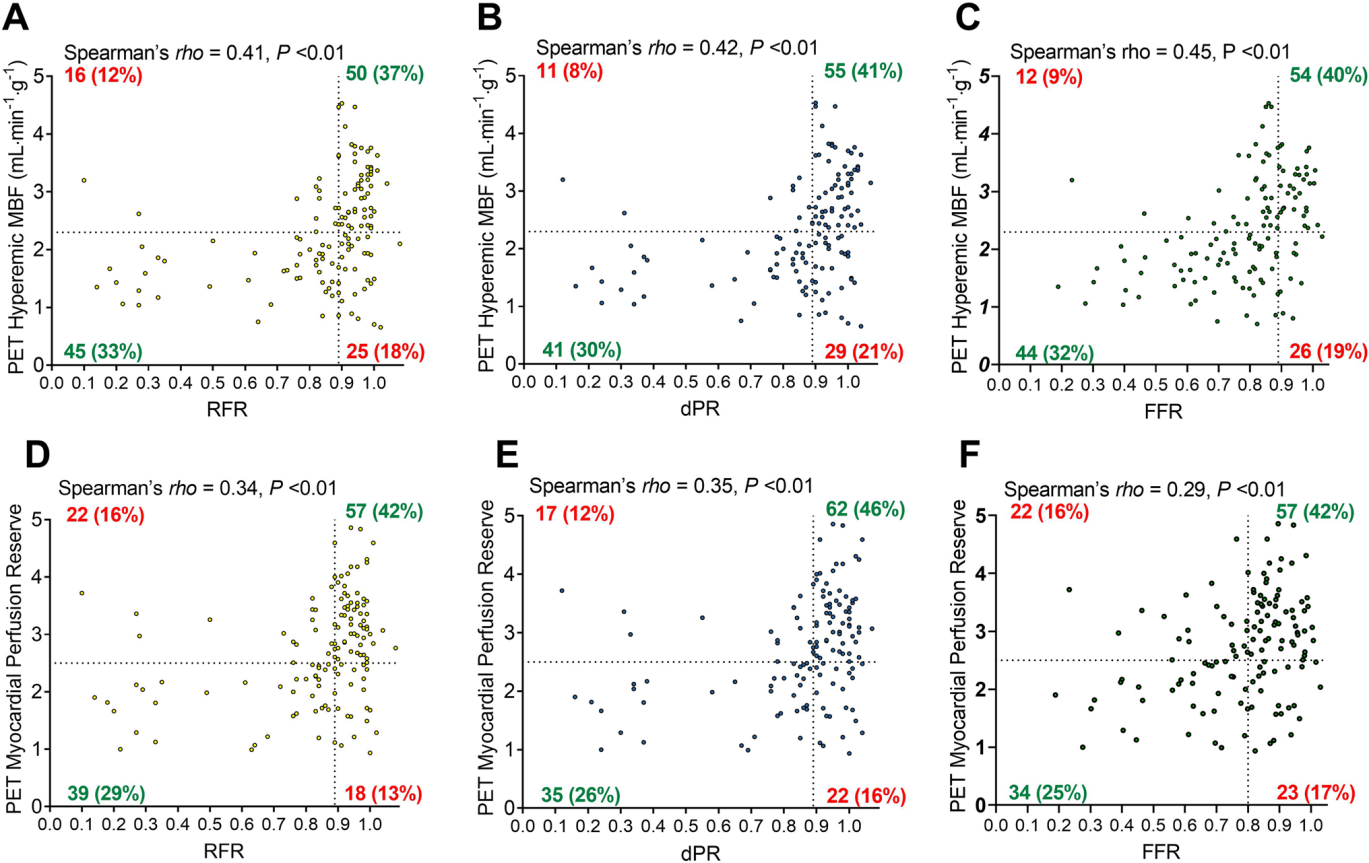
Correlation between physiologic indices and PET values, only stenoses included (*N* = 136). Scatter plots shows correlations between the different pressure indices and **A**–**C** PET hMBF and **D**–**F** MPR in stenoses group. Spearman’s rho reported in each graphic. *PET* Positron emission tomography, *RFR *resting full-cycle ratio, *dPR *diastolic pressure ratio, *FFR *fractional flow reserve

Positive moderate correlations among RFR, dPR, and FFR with [^15^O]H_2_O regional PET hMBF and MPR were found, as shown in Fig. 3. FFR values showed a high correlation with RFR and dPR (Spearman rho; 0.88 and 0.89, respectively) (Online Resource 1).

**Fig. 4:**
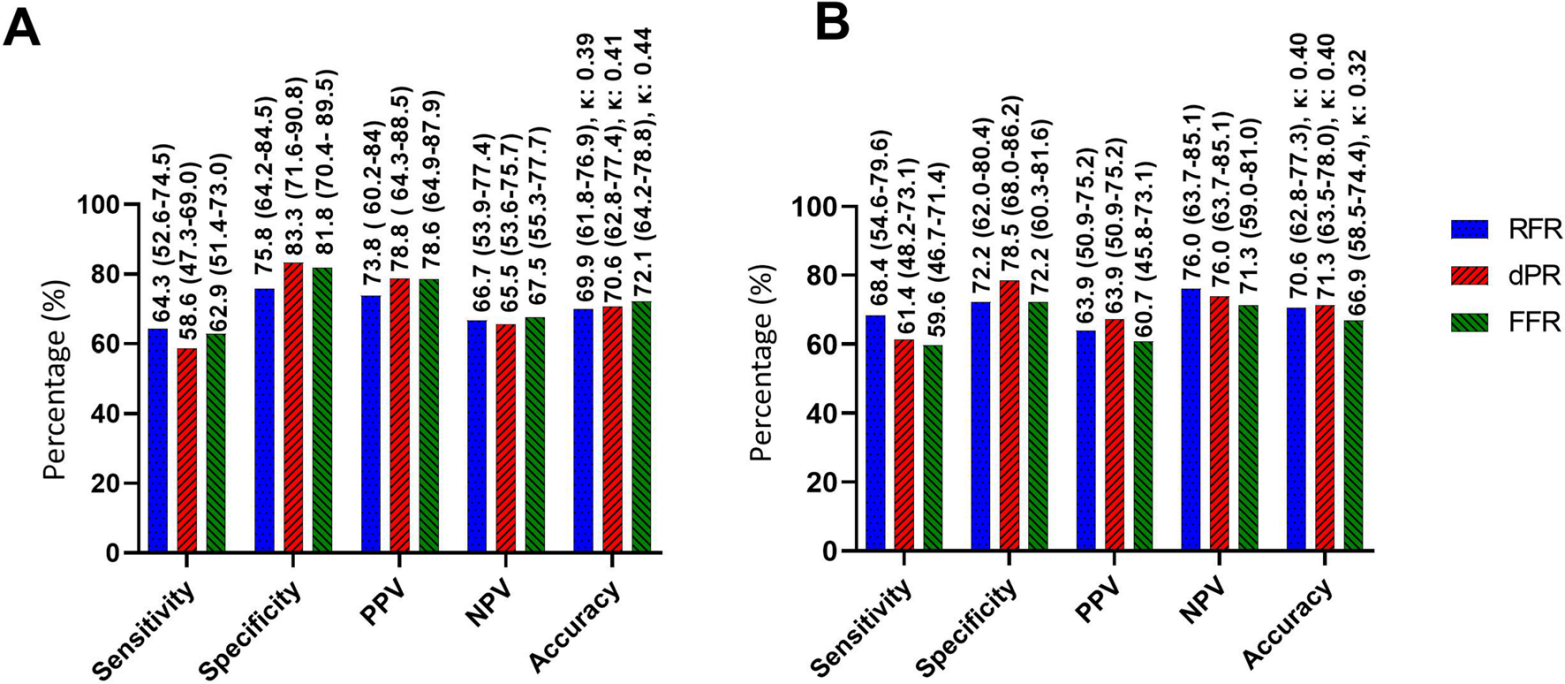
Diagnostic performance of physiological indices, only stenoses included (*N* = 136). Diagnostic performance of RFR, dPR, and FFR using **A** PET hMBF and **B** MPR as reference standards for regional myocardial ischemia in coronary stenoses. Sensitivity, specificity, PPV, NPV, and diagnostic accuracy are reported with their respective 95% confidence interval. Cohen’s kappa coefficient is shown. Significance testing was performed between the test accuracy of each pressure indices; *p* values are shown only if < 0.05. *PET *Positron emission tomography, *RFR *resting full-cycle ratio, *dPR *diastolic pressure ratio, *FFR *fractional flow reserve, *hMBF *hyperemic myocardial blood flow, *MPR *myocardial perfusion reserve, *PPV* positive predictive value, *NPV* negative predictive value

Figure 4 shows the diagnostic characteristics of RFR, dPR, and FFR for predicting regional ischemia based on PET hMBF and MPR. With hMBF as the reference, the sensitivity, specificity, positive predictive value, negative predictive value, and diagnostic accuracy of RFR were 64% (95% CI 52–75), 76% (95% CI 64–85), 74% (95% CI 60–84), 67% (95% CI 54–77), and 70% (95% CI 62–77), respectively. Those of dPR were 59% (95% CI 47–69), 83%, (95% CI 72–91), 79% (95% CI 64–89), 66% (95% CI 54–76), and 71% (95% CI 63–77), respectively.

We did not find differences in diagnostic accuracies between the ratios when we used PET hMBF (RFR vs. dPR, *p* = 0.93; RFR vs. FFR, *p* = 0.77; and for dPR vs. FFR, *p* = 0.63) and MPR (RFR vs. dPR, *p* = 0.93; RFR vs. FFR, *p* = 0.85 and for dPR vs. FFR, *p* = 0.78) as reference.

**Fig. 5:**
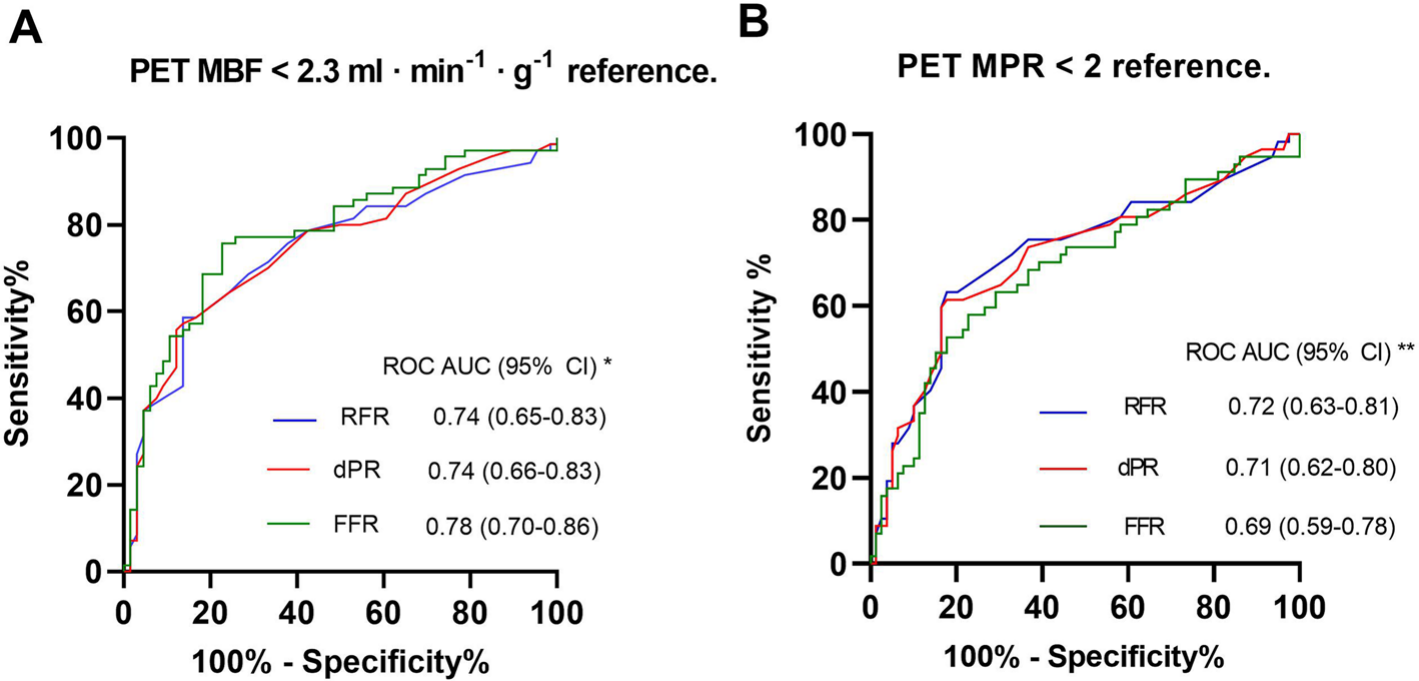
ROC Analysis using PET-derived parameters as the reference standard, only stenosis included. **A** ROC curves for RFR, dPR, and FFR predicting PET hMBF in the stenoses group and their respective AUCs are presented. **B** ROC curves for physiological indices are shown using PET MPR as reference. *ROC* receiver operating characteristic, *PET *positron emission tomography, *RFR *full-cycle ratio, *dPR *diastolic pressure ratio, *FFR *fractional flow reserve, *MBF *myocardial blood flow, *MPR *myocardial perfusion reserve, *AUC *area under the curve. * There were non-significant differences between the ROC AUC values of the indices using on De Long Test (RFR vs dPR, *p* = 0.91; RFR vs FFR, *p* = 0.5; and dPR vs FFR, *p* = 0.56). ** There were non-significant differences between the ROC AUC values of the indices using on De Long Test (RFR vs dPR, *p* = 0.93; RFR vs FFR, *p* = 0.54; and dPR vs FFR, *p* = 0.59)

Figure 5 shows the ROC curves for the physiological ratios to predict PET hMBF. For RFR the ROC AUC was 0.74 (95% CI 0.65–0.83), for dPR 0.74 (95% CI 0.66–0.83), and 0.78 (95% CI 0.70–0.86) for FFR. No significant differences were found between the areas under the curve of all the tested indices. Additionally, no differences were found when PET MPR was used as reference standard.

### Physiological parameters and positron emission tomography in all vessels

Online Resource 2 shows the diagnostic test characteristics for the dichotomized physiological ratios compared with PET hMBF as the reference standard. In the 320 vessels tested the diagnostic accuracy was 75% (95% CI 69–80) for RFR, 75% (95%CI 69–81) for dPR, and 77% (95% CI 71–82) for FFR. Similar results were found when we used MPR as the reference. The ROC analysis for RFR, dPR, and FFR showed AUC of 0.72, 0.73, and 0.74, respectively, for detecting abnormal PET hMBF, with no significant differences between them (RFR vs dPR, *p* = 0.92; RFR vs FFR, *p* = 0.62; and dPR vs FFR, *p* = 0.69).

### Discrepancies between FFR and NHPR

**Fig. 6:**
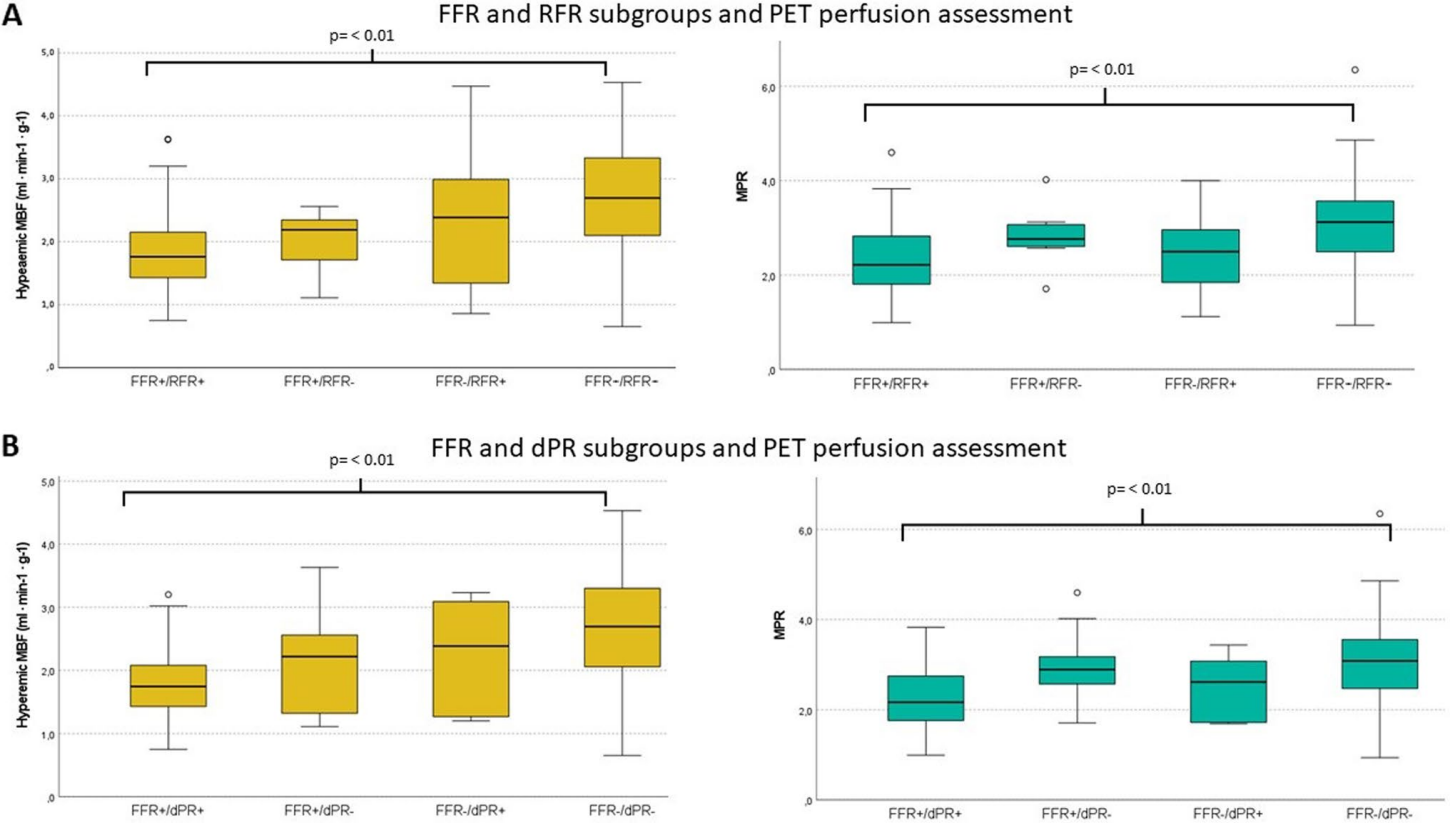
PET myocardial perfusion in FFR and NHPR subgroups. Boxplot represents the hMBF and the MPR in the different subgroups based on the pressure indices. Significance testing was performed between each subgroup; *p* values are shown only if < 0.05. *PET *Positron emission tomography, *RFR* resting full-cycle ratio, *dPR* diastolic pressure ratio, *FFR *fractional flow reserve, *MBF *myocardial blood flow, *MPR *myocardial perfusion reserve

Of the 136 coronary stenoses assessed, 19 (14%) exhibited discordance between binary defined FFR and RFR. There was no difference in clinical characteristics between the FFR/RFR groups, as documented in Online Resource 3. When assessing angiographic data across these groups, the positive concordant (FFR + /RFR +) had on average a higher lesion diameter stenosis than negative concordant groups (difference: 18.2% (95% CI 12.3 to 24.0); *p* < 0.01). In terms of PET perfusion metrics, FFR + /RFR + patients had significantly lower values than the FFR −/RFR − group. This was consistently observed for hMBF (difference: -0.83 (95% CI − 1.24 to − 0.43); *p* < 0.01) and MPR (difference: − 0.874 (95% CI − 1.19 to − 0.29); *p* < 0.01) as depicted in Fig. 6 A. The hMBF and MPR from the discordant groups did not differ from the concordant positive and negative groups, nor within themselves. FFR and RFR values from the discordant groups were numerically closer to their cutoff value (Online Resource 3). When grouping by the results of FFR and dPR we found 16 (12%) discordant cases, and subgroup characteristics are displayed in Online Resource 4 and Fig. 6 B. Additionally, a detailed report of the angiographic characteristics and PET regional perfusion for each of the discordant FFR/NHPR stenoses is provided in Online Resource 5.

### Additional diagnostic value of NHPR over FFR for detecting flow limiting stenosis

Univariable analysis showed that FFR, RFR, and dPR are individual significant predictors of regional myocardial ischemia defined by PET hMBF. In a multivariable analysis with only FFR in the base model, RFR and dPR were no longer significant predictors of hMBF (Table 3). The addition of RFR or dPR to a model including FFR did not significantly increase the diagnostic information provided to identify PET hMBF (Online Resource 6).

**Table 3:** Univariable and multivariable analyses of the performance of different pressure ratios to predict ischemia based on hyperemic MBF

Model	Univariable analysis		Multivariable analysis*	
	Odds ratio (95% CI)	*p* value	Odds ratio (95% CI)	*p* value
FFR	7.62 (3.4–17.02)	< 0.001	–	–
RFR	5.63 (2.63–12.01)	< 0.001	2.08 (0.74–5.83)	0.16
dPR	7.07 (3.13–15.99)	< 0.001	2.71 (0.87–8.47)	0.09

*The multivariable analysis incorporated only the binary FFR in the base model

*FFR *fractional flow reserve, *RFR *resting full-cycle ratio, *dPR *diastolic pressure ratio

## Discussion

In the present study, we compared the diagnostic performance of two newer NHPR (RFR and dPR) with the established FFR for the prediction of myocardial ischemia defined by [^15^O]H_2_O PET perfusion. The principal findings of this study are as follows: (1) The diagnostic performance of RFR, dPR, and FFR for detecting flow limiting coronary stenosis defined by regional PET quantitative perfusion was not significantly different. (2) The discordance rate between FFR and RFR/dPR is between 12 and 14%. (3) The diagnostic capacity of FFR to detect myocardial ischemia is not modified when combining its use with RFR or dPR.

### Validation of pressure indices

Our results show that the diagnostic accuracies of RFR, dPR, and FFR for detecting regional myocardial ischemia are approximately 70% when using an independent reference standard. Notably, no significant disparities in this metric were observed among these ratios (Fig. 4). These results are consistent with prior literature findings, which document a diagnostic accuracy between 66 and 76% when comparing FFR or others non-hyperemic coronary pressure ratios against PET-derived hMBF, MPR, or a combination of these parameters. [5, 6, 9] Based on this evidence, we could consider FFR, RFR, and dPR as equivalent methods for invasively detecting hemodinamically significant lesions.

### Exploring differences between FFR and NHPR

When comparing the FFR to NHPR, we found a discordance rates between FFR and RFR of 14% and 12% for dPR (Online Resource 1), similar to findings of previous research. [6, 18–20]

In our work, both discordant groups had an hMBF and MPR that was not significantly different than the values from the concordant positive group. However, it is noteworthy that the FFR + /NHPR − patients presented a numerically higher MPR, compared to both FFR + /NHPR + and FFR −/NHPR + groups; this observation was present for RFR and dPR. This findings are in line with previous evidence, indicating that the FFR + /NHPR − subset typically demonstrates higher flow reserves than the concordant positive group, quantified invasively or with ^13^N-ammonia PET perfusion. [18, 23, 24] This group could represents patients with epicardial coronary disease but with an adequate relative flow increase in response to hyperemic agents, indicative of a functionally preserved microcirculation.

Interestingly, the other discordant group (FFR −/NHPR +) had similar hMBF and MPR than the positive concordant cases. In this group the prevalence of diabetes, hypertension and dyslipidemia it’s similar to the group with concordant positive pressure ratios, but numerically higher than those from the FFR + /NHPR − group. Such observations align with the proposition that this subgroup might predominantly encompass patients presenting diffuse epicardial disease or small vessel disease, with subsequent impaired hyperemic response to vasodilator stress [25, 26].

### Integrated use of FFR with NHPR

Recent registry reports have shown that the combined use of FFR with NHPR could be useful in clinical practice, since discordant FFR/NHPR lesions without revascularization hold an unfavorable prognosis, similar to patients with positive concordant values [20]. In this study we tested if the combined use of FFR and NHPR could increase the diagnostic precision to predict regional myocardial ischemia by PET quantitative perfusion. Our results showed that adding binary RFR (or dPR) to FFR did not increase the diagnostic information provided by the stand alone FFR strategy. Since severely symptomatic patients with low myocardial perfusion are the theoretical group that could most benefit from revascularization for controlling ischemic symptoms, our results suggest that the employment of RFR (or dPR) could be a reasonable alternative to the use of FFR in light of their equivalent efficacy in ischemia detection [27, 28].

### Study limitations

Some limitations should be acknowledged. First, the calculation of the pressure ratios was performed retrospectively. However, since the analysis was performed by an automated algorithm and carried out by blinded researchers, we believe external validity is not hampered by this potential limitation. Secondly, we used a predefined binary cutoff for our gold standard and also for the pressure indices. Myocardial blood flow is a continuous parameter, that has described variability between different individuals [29]. A specific value of hMBF or MPR could be adequate to meet the myocardial demands of a patient, but for others this same myocardial blood flow could be under the ischemic threshold. However, decision making in clinical practice imposes dichotomous definitions for practical reasons. With this in mind, we carefully selected the cutoff values based on the previous literature [6, 12, 13, 16]. Third, excluding high-risk patient with previous history of myocardial infarction, revascularization, and subtotal stenoses hinders the implement of our results to these specific scenarios. Fourth, the low number of discordant cases found in this trial limited the power for detecting differences in clinical characteristic and perfusion metrics between the FFR/NHPR groups. Importantly, the values obtained were in line with other studies, providing support to the limited evidence available.

## Conclusions

In the present study, the novel non-hyperemic pressure ratios RFR and dPR performed similarly to FFR for detecting regional myocardial ischemia when using the non-invasive gold standard of quantitative myocardial perfusion as the reference standard. This finding suggests that RFR and dPR may serve as a viable alternative hyperemic-free strategy to FFR for guiding revascularization in symptomatic patients with coronary artery disease. Furthermore, the integration of FFR with these NHPR does not enhance the diagnostic capability for detecting myocardial ischemia. Differences in clinical and myocardial perfusion characteristics between the discordant FFR and NHPR groups should be explored further in the future.

### Supplementary Information

Below is the link to the electronic supplementary material.

Supplementary file1 (PDF 995 KB)

## Abbreviations

PCI: Percutaneous coronary intervention
FFR: Fractional flow reserve
iFR: Instantaneous wave-free ratio
NHPR: Non-hyperemic pressure ratio
PET: Positron emission tomography
RFR: Resting full-cycle ratio
dPR: Diastolic pressure ratio
ROC: Receiver operating characteristic
hMBF: Hyperemic myocardial blood flow
MPR: Myocardial perfusion reserve
AUC: Area under the curve
