# Supplementary material for: Validation of resting full-cycle ratio and diastolic pressure ratio with [15O]H2O positron emission tomography myocardial perfusion
Source: Heart Vessels. 2024 Feb 17;39(4):299–309. doi: 10.1007/s00380-023-02356-4 (PMC10920410; doi:10.1007/s00380-023-02356-4)
Supplement: Supplementary file 1 — Supplementary file1 (PDF 995 KB) [file 380_2023_2356_MOESM1_ESM.pdf]

Supplementary Material

**Title:** Validation of resting full-cycle ratio and diastolic pressure ratio with [$^{15}O$]$H_2O$ positron emission tomography myocardial perfusion.

**Journal:** Heart and Vessels

**List of authors:** Jorge Dahdal MD ; Frank Bakker MD, Johan Svanerud, Ibrahim Danad MD PhD, Roel S Driessen MD, Pieter G Raijmakers MD PhD, Hendrik J Harms MD PhD, Adriaan A Lammertsma PhD, Tim P van de Hoef MD PhD, Yolande Appelman MD PhD, Niels van Royen MD PhD, Paul Knaapen MD PhD, Guus A de Waard MD PhD.

**Corresponding Author**

Guus A de Waard MD PhD

Department of Cardiology, Amsterdam University Medical Center, De Boelelaan 1117, 1081 HV Amsterdam, The Netherlands.

g.dewaard@amsterdamumc.nl

Online Resource 1

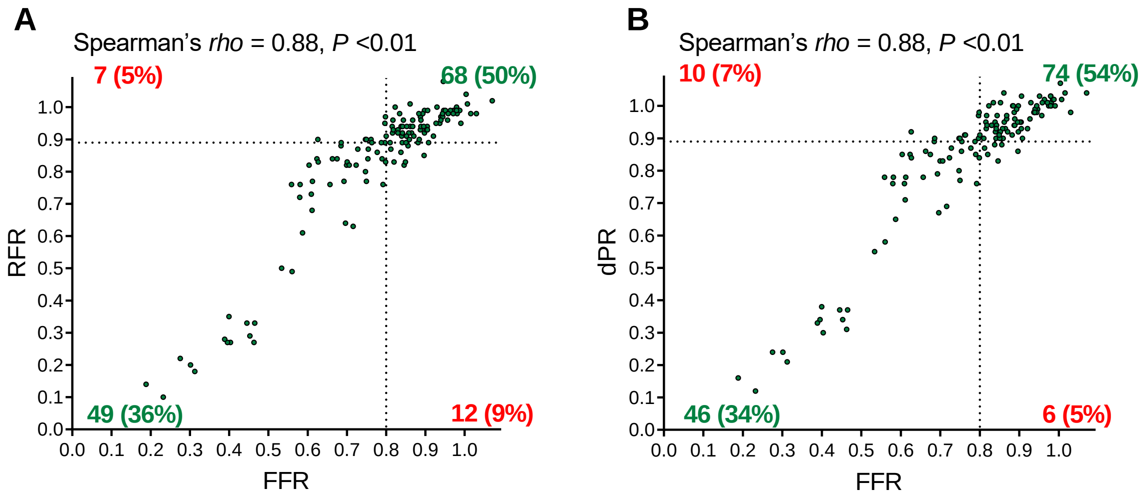


**Correlation between hyperemic and non-hyperemic physiologic indices, only stenosis included (n = 136).** Scatter plot showing correlation between A) RFR vs FFR and B) dPR vs FFR. Spearman's rho reported in each graphic. Pressure physiological indices expressed in unitless numbers. PET=Positron emission tomography; RFR = resting full-cycle ratio; dPR = diastolic pressure ratio; FFR = fractional flow reserve.

Online Resource 2

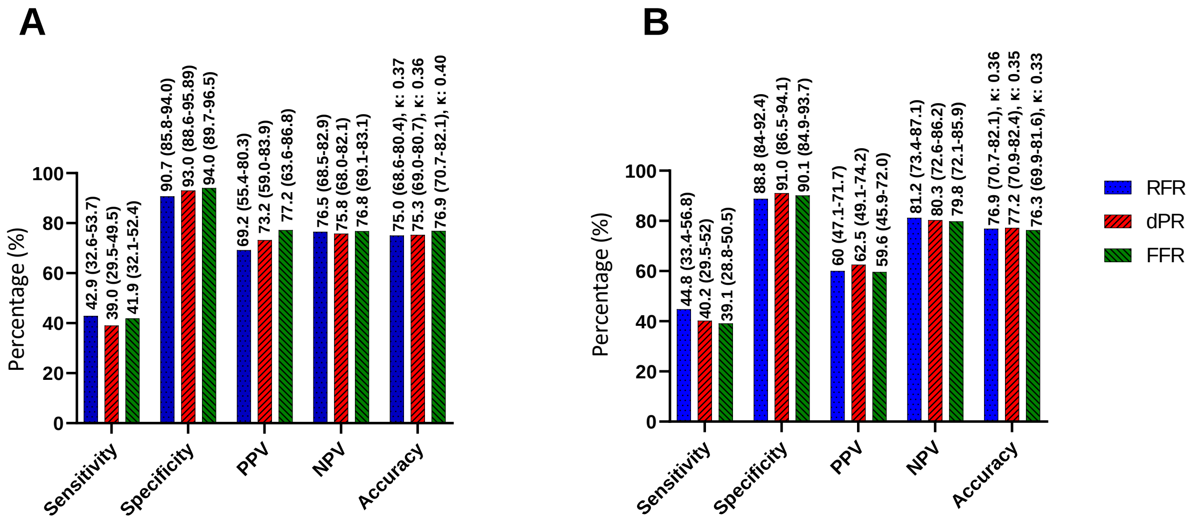


**Diagnostic performance of physiological indices, all vessels included (N= 320).** Diagnostic performance of RFR, dPR, FFR using A) PET hMBF and B) MPR as reference standards for predicting myocardial ischemia in all the vessels studied. Sensitivity, Specificity, PPV, NPV and Accuracy are reported with their respective 95% confidence interval. Cohen's kappa coefficient is shown. Significance testing was performed between the test accuracy of the different physiological indices , p-values are shown only if <0.05.

ROC = receiver-operating characteristic. PET=Positron emission tomography RFR = resting full-cycle ratio; dPR = diastolic pressure ratio; FFR = fractional flow reserve; hMBF = hyperemic myocardial blood flow; MPR = myocardial perfusion reserve; AUC = area under the curve.

Online Resource 3

Patient characteristics, angiography and PET results based on FFR and RFR groups.

| | Group 1 (FFR+/ RFR+) | Group 2 (FFR+/ RFR-) | Group 3 (FFR-/ RFR+) | Group 4 (FFR-/ RFR-) | Overall p value |
|---|---|---|---|---|---|
| **N of vessels with stenoses** | 49 | 7 | 12 | 68 | |
| **Male Sex (%)** | 88 | 100 | 58 | 67 | 0.54 |
| **Diabetes mellitus (%)** | 29 | 20 | 38 | 11 | 0.13 |
| **Hypertension (%)** | 59 | 40 | 57 | 56 | 0.85 |
| **Dyslipidemia (%)** | 43 | 40 | 43 | 38 | 0.88 |
| **Body mass index (BMI, kg/m2)** | 27.7 (2.9) | 25.4 (3.1) | 25.9 (2.7) | 26.5 (3.1) | 0.17 |
| **Angiography** | | | | | |
| **Lesion location = LAD (%)** | 61.2 | 57.1 | 41.7 | 42.6 | 0.36 |
| **Mean diameter stenosis % (SD)** | 64.29 (12.9)* | 54.78 (10.8) | 55.12 (15.5) | 46.11 (9.8)* | <0.01 |
| **PET** | | | | | |
| **Hyperemic MBF (SD) ($ml \cdot min^{-1} \cdot g^{-1}$)** | 1.85 (0.65)* | 1.99 (0.56) | 2.30 (1.06) | 2.69 (0.88)* | <0.01 |
| **MPR (SD)** | 2.31 (0.80)* | 2.83 (0.69) | 2.50 (0.81) | 3.06 (0.97)* | <0.01 |
| **Intracoronary pressure** | | | | | |
| **FFR** | 0.59 (0.16) | 0.75 (0.06) | 0.85 (0.03) | 0.91 (0.06) | <0.01 |
| **RFR** | 0.64 (0.25) | 0.91 (0.03) | 0.86 (0.03) | 0.96 (0.04) | <0.01 |

The p value comparing Group 1 vs Group 4 diameter stenosis, MBF and MPR was <0.01.

RFR = resting full-cycle ratio; FFR = fractional flow reserve; LAD= left anterior descending;

SD= standard deviation; PET= positron emission tomography; MBF = myocardial blood flow;

MPR = myocardial perfusion reserve.

Online Resource 4

Patient characteristics, angiography and PET results based on FFR and dPR groups.

| | Group 1 (FFR+/ dPR+) | Group 2 (FFR+/ dPR-) | Group 3 (FFR-/dPR+) | Group 4 (FFR-/ dPR-) | Overall p value |
|---|---|---|---|---|---|
| **N of vessels with stenoses** | 46 | 10 | 6 | 74 | |
| **Male Sex (%)** | 95 | 86 | 100 | 85 | 0.12 |
| **Diabetes mellitus (%)** | 31 | 29 | 50 | 16 | 0.20 |
| **Hypertension (%)** | 60 | 43 | 75 | 54 | 0.83 |
| **Dyslipidemia (%)** | 43 | 43 | 75 | 35 | 0.48 |
| **Body mass index (BMI, kg/m2)** | 27.5 (2.7) | 27.1 (4.5) | 26.6 (3.4) | 26.4 (3.04) | 0.49 |
| **Angiography** | | | | | |
| **Lesion location = LAD (%)** | 60.9 | 60.0 | 50.0 | 41.9 | 0.44 |
| **Mean diameter stenosis % (SD)** | 65.68 (12.1)* | 51.25 (10.5) | 55.5 (18.3) | 46.8 (10.3)* | <0.01 |
| **PET** | | | | | |
| **Hyperemic MBF (SD) (ml · $min^{-1}$ · $g^{-1}$)** | 1.79 (0.54)* | 2.24 (0.89) | 2.26 (0.89) | 2.66 (0.92)* | <0.01 |
| **MPR (SD)** | 2.24 (0.74)* | 3.00 (0.81) | 2.52 (0.72) | 3.01 (0.97)* | <0.01 |
| **Intracoronary pressure** | | | | | |
| **FFR** | 0.59 (0.16) | 0.75 (0.06) | 0.84 (0.3) | 0.91 (0.06) | <0.01 |
| **dPR** | 0.65 (0.25) | 0.92 (0.03) | 0.86 (0.02) | 0.97 (0.04) | <0.01 |

The p value comparing Group 1 vs Group 4 diameter stenosis, MBF and MPR was <0.01.

dPR = diastolic pressure ratio; FFR = fractional flow reserve; LAD= left anterior descending; SD= standard deviation; PET= positron emission tomography; MBF = myocardial blood flow; MPR = myocardial perfusion reserve.

Online Resource 5

Detailed description of individual discordant FFR/NHPR stenoses, PET regional quantitative perfusion and angiographic data.

| FFR+/NHPR- (RFR or dPR) | | | | | | | | | |
|---|---|---|---|---|---|---|---|---|---|
| | **Age** | **Vessel** | **DS%** | **Location** | **FFR** | **RFR** | **dPR** | **PET hMBF** | **PET MPR** |
| 1 | 63 | CX | 49.0 | Distal | **0.80** | 0.95 | 0.97 | **2.19** | 3.01 |
| 2 | 63 | RCA | 58.5 | Proximal | **0.76** | 0.90 | 0.91 | 2.44 | 2.57 |
| 3 | 50 | RCA | 58.5 | Mid | **0.80** | 0.97 | 0.98 | **1.32** | 2.64 |
| 4 | 45 | LAD | 56.0 | Mid | **0.75** | 0.90 | 0.90 | 2.56 | 3.12 |
| 5 | 52 | LAD | 40.0 | Mid | **0.80** | 0.91 | 0.91 | 2.25 | 4.02 |
| 6 | 66 | LAD | 74.0 | Mid | **0.63** | 0.90 | 0.92 | **1.11** | 1.71 |
| 7 | 65 | LAD | 47.5 | Proximal | **0.75** | 0.90 | 0.90 | **2.10** | 2.76 |
| 8 | 44 | RCA | 43.0 | Proximal | **0.69** | **0.89** | 0.90 | **1.25** | **2.40** |
| 9 | 59 | LAD | 42.0 | Distal | **0.80** | **0.89** | 0.90 | 3.62 | 3.18 |
| 10 | 65 | RI | 44.0 | Distal | **0.76** | **0.89** | 0.91 | 3.63 | 4.59 |
| **FFR-/NHPR+ (RFR or DPR)** | | | | | | | | | |
| | **Age** | **Vessel** | **DS%** | **Location** | **FFR** | **RFR** | **dPR** | **PET hMBF** | **PET MPR** |
| 1 | 48 | RCA | 59.0 | Proximal | 0.81 | **0.87** | **0.87** | **1.20** | **1.69** |
| 2 | 68 | LAD | 71.5 | Mid | 0.86 | **0.88** | **0.88** | 2.72 | 2.84 |
| 3 | 63 | CX | 48.5 | Mid | 0.82 | **0.83** | **0.85** | 3.23 | 3.08 |
| 4 | 65 | LAD | 43.0 | Mid | 0.85 | **0.82** | **0.83** | 3.09 | 3.43 |
| 5 | 73 | CX | 80.0 | Mid | 0.90 | **0.85** | **0.86** | **1.27** | **1.72** |
| 6 | 67 | LAD | 31.0 | Mid | 0.84 | **0.86** | **0.88** | **2.05** | **2.38** |
| 7 | 80 | RCA | 50.0 | Mid | 0.91 | **0.89** | 0.90 | 2.72 | 2.84 |
| 8 | 52 | CX | 62.5 | Proximal | 0.84 | **0.89** | 0.90 | **1.42** | **1.97** |
| 9 | 60 | CX | 64.0 | Mid | 0.88 | **0.89** | 0.91 | **0.86** | **1.12** |
| 10 | 53 | CX | 69.0 | Mid | 0.81 | **0.89** | 0.90 | **1.69** | 2.61 |
| 11 | 61 | LAD | 53.0 | Proximal | 0.85 | **0.89** | 0.90 | 4.47 | 4.00 |
| 12 | 50 | LAD | 30.0 | Mid | 0.85 | **0.83** | 0.91 | 2.89 | **2.28** |

Abnormal values of pressure indices or perfusion metric were are highlighted with bold font. hMBF expressed in ml · $min^{-1}$ · $g^{-1}$**.** FFR = fractional flow reserve; NHPR= non-hyperemic pressure ratio; FFR = fractional flow reserve; RFR= resting full-cycle ratio; dPR = diastolic pressure ratio; LAD= left anterior descending; RCA= right coronary artery; CX = circumflex

artery;  PET = positron emission tomography; hMBF = hyperemic myocardial blood flow; MPR = myocardial perfusion reserve.

Online Resource 6

Logistic regression models including pressure indices an their combination for predicting hyperemic MBF.

| Models | -2LL | LRT (p value) |
|---|---|---|
| **FFR** | 608.7 | - |
| **FFR + RFR** | 607.8 | 0.34 |
| **FFR + dPR** | 608.6 | 0.75 |

LRT was performed to compare the initial model (FFR) with the model with the addition of both RFR and dPR separately. -2LL = - 2 (log likelihood); LRT = likelihood ratio test; RFR = resting full-cycle ratio; dPR = diastolic pressure ratio.
